# Proliferation and metabolic activity of the Atlantic sturgeon cell line AOXlar7y under short-term serum-reduced conditions, and the effect of stimulation with growth factors and cytokines

**DOI:** 10.3389/ftox.2025.1636776

**Published:** 2025-10-01

**Authors:** Valeria Di Leonardo, Julia Brenmoehl, Heike Wanka, Bianka Grunow

**Affiliations:** ^1^ Fish Growth Physiology, Research Institute for Farm Animal Biology (FBN), Dummerstorf, Germany; ^2^ Endocrinology of Farm Animals, Research Institute for Farm Animal Biology (FBN), Dummerstorf, Germany; ^3^ Institute of Physiology, University Medicine, Greifswald, Germany

**Keywords:** LIF, FGF-2, IFN-γ, IGF-1, interleukin, fish cell line, xCELLigence, seahorse

## Abstract

**Introduction:**

Fish cell lines represent a powerful tool for studying the biology and toxicology of aquatic species in compliance with the 3Rs principles. In addition, they hold potential for more advanced biotechnological applications. However, fish cell cultures are mainly cultivated with fetal bovine serum. Therefore, in this study, we investigated the impact of serum reduction and the effects of six growth factors and cytokines on a sturgeon larval cell line (AOXla7y), which has been previously proven to be a valuable model for climate change and toxicology studies.

**Methods:**

The serum reduction (from 10% to 5% and 2%) and the addition of two concentrations (10 and 50 ng/mL) of six growth factors and cytokines (FGF-2, IGF-1, LIF, IFN-γ, IL-13, and IL-15) to the 2% serum growth medium were evaluated over 6 days of cultivation. The morphology and cell density were determined using phasecontrast images after the experiment ended, while real-time label-free cell impedance (xCELLigence) was recorded throughout the cultivation period. Moreover, the end-point oxygen consumption in basal and uncoupled respiration conditions was analyzed with the Seahorse XF Cell Mito Stress Test Kit.

**Results:**

The results demonstrated a general adaptation of the sturgeon cell line to a serum-reduced environment and the modulatory effects of growth factor and cytokine supplementation on cell growth and metabolism.

**Discussion:**

These findings suggest that the sturgeon cell line has the potential to transition to a serumfree medium without major observed morphological modifications and with a limited reduction in proliferation. Its metabolism was differentially modulated by the signaling of growth factors and cytokines and exhibited a variable metabolic phenotype under mitochondrial stress. This study provides a characterization of the Atlantic sturgeon cell metabolism and offers a preliminary assessment for developing an animal-free and chemically defined medium.

## 1 Introduction

Fish cell cultures have become a relevant model for basic research and biotechnological applications. They offer an alternative to whole animal experiments for exploring physiological, pathological, biochemical, genetic, and molecular mechanisms in a fast, standardized, and high-throughput setting (Zurita et al., 2019; Goswami et al., 2022a; He et al., 2023). In recent years, a growing interest in utilizing fish cell cultures for industrial applications, particularly in the development of cell-based meat, has led to several rounds of public and private investments intending to advance alternative food systems, ultimately reducing the overexploitation of animals and the environment (Goswami et al., 2022b; Goswami et al., 2023). Both basic research and industrial applications of fish cell lines converge on the need to transition to animal-free media. This is motivated, on the one hand, by ethical reasons and the need for a more standardized medium, and on the other hand, to achieve sustainable and scalable solutions for industrial applications. Despite their differences from mammals, fish cells are also typically cultured with a basic medium supplemented with fetal bovine serum (FBS), also referred to as fetal calf serum (FCS). This provides a complex mix of nutrients essential for cell subsistence (van der Valk et al., 2010; Lakra et al., 2011; Rubio et al., 2019).

However, FBS is known to have several limits when used routinely for cell propagation and experiments: the ethical issues arising from its production, the costs, the risk of contamination, the lack of chemically defined components and the batch-to-batch variability make the need for this medium supplement to be substituted in the near future (van der Valk et al., 2010; Gstraunthaler et al., 2013; Liu et al., 2023). While alternatives are emerging for mammalian cell cultures, suitable serum-free options for fish cells remain scarce and under-explored (Volff, 2005).

Serum contains a complex array of proteins, hormones, growth factors (GFs), cytokines (CKs), lipids, carbohydrates, vitamins, minerals, and other elements. To develop serum-free media, researchers have been investigating alternative sources to mimic FBS, evaluating the suitability of both undefined and defined solutions (Lee et al., 2022a). The resulting culture media can be categorized into the following groups: serum-free, protein-free, animal-free, and chemically defined. Serum-free media focus on the specific elimination of FBS. Protein-free media are elaborated to avoid using high-molecular-weight proteins and protein fractions. Animal-free media are made without any animal component, while chemically defined media are designed to prevent any element with an unknown composition. Above all, when available, animal-free chemically defined media are preferred to eliminate the drawbacks associated with serum use as indicated by the Good Cell Culture Practices (GCCP) (van der Valk et al., 2010; Jacobs et al., 2023).

In this field, hormones, GFs, and CKs are particularly studied for their central role in cellular signaling and their potential to be included in chemically defined media (Gross and Rotwein, 2017; Goswami et al., 2022b). Although their tissue specificity and complex role in many cellular pathways, some GFs exhibit mitogenic effects across various cell types, often in a concentration-dependent manner (Jones and Kazlauskas, 2001; Goswami et al., 2022b). As a result, multiple studies have focused on incorporating GFs into culture media, starting with one of the first works by Hayashi and Sato in 1976, which demonstrated that hormones could be used to replace serum (Hayashi and Sato, 1976; van der Valk et al., 2010; Venkatesan et al., 2022). CKs are generally studied for their role in modulating immune response, but they are increasingly recognized as key players in cell proliferation and developmental processes (Zolti et al., 1991; Oppenheim, 2003; Idrees et al., 2020). A key advantage of GFs and CKs is that they can be produced through biotechnological approaches using microbial or mammalian cell factories (Tschofen et al., 2016; Gifre et al., 2017). However, their practical use heavily depends on species-specific biology, cell line characteristics, and optimal dosage, underlying the need for long and extensive studies to develop custom-made solutions (Venkatesan et al., 2022; Jacobs et al., 2023).

Despite these challenges, chemically defined media supplemented with recombinant GFs and CKs can offer a more precise, reproducible, and ethical alternative to FBS in various research fields ranging from biomedical research (Ren et al., 2020; Lee et al., 2022b) to cellular agriculture (Shaikh et al., 2021). Nevertheless, their environmental life-cycle assessment is still under discussion for larger industrial scales (Urbischek et al., 2019).

To date, several serum-free chemically defined media using GFs and CKs have been tested (Yao and Asayama, 2017; Urbischek et al., 2019; Rafnsdóttir Ó et al., 2023; Skrivergaard et al., 2023) or are already commercially available (Badenes et al., 2016), eliciting promising results in terms of cell maintenance and proliferation in different kinds of cells, including stem cell cultures (Kolkmann et al., 2022). Fewer examples of serum-free media have been tested in fish cell cultures, but the explored alternatives are generally undefined. For example, Radošević et al. successfully adapted the permanent fish cell line CCO (channel catfish ovary) in a commercially available serum-free medium known as UltraCulture SFM (UC SFM) purchased from Lonza (Verviers, Belgium) and tested three protein hydrolysates to assess their role in cell proliferation (Radošević et al., 2016). Although commercially available serum-free media have yielded promising results, they are typically supplemented with a proprietary and undisclosed combination of GFs. To avoid these confounding variables, media such as Leibovitz-15 media (L-15) basal medium are ideal, as their composition is completely defined and transparent, and no exogenous growth factors are added.

The need to develop animal-free *in vitro* systems encompasses the field of toxicology, where fish cell lines offer a promising alternative to animal testing for chemical risk assessments. The recent inclusion of the RTgill-W1 cell line assay (OECD TG 249) in the OECD guidelines and in the ISO guidelines (ISO 21115:2019 Water quality - Determination of acute toxicity of water samples and chemicals) represents a progressive step toward reducing animal use in toxicology (Fischer et al., 2019). This rainbow trout gill cell line (Bols et al., 1994) has significantly reduced the need for conducting acute toxicity tests on live fish, owing to its strong predictive capacity of *in vivo* outcomes.

Within this study, the serum reduction and two concentrations of six recombinant GFs and CKs (FGF-2, IGF-1, LIF, IFN-γ, IL-13, and IL-15) were tested on the Atlantic sturgeon whole larval cell line AOXlar7y. The selection of signaling molecules was based either on their well-established mitogenic effect in fish cells or on emerging evidence supporting their role in regulating tissue homeostasis, with a focus on early developmental stages. This focus aligns with the larval origin of the AOXlar7y cell line. The Fibroblast Growth Factor 2 (FGF-2) is considered mitogenic and is commonly supplemented in fish cell culture media (Lakra et al., 2011). Leukemia Inhibitory Factor (LIF) was considered in its role in promoting self-renewal of embryonic stem cells in teleosts (Wei et al., 2018), and is generally added to the media of newly isolated embryonic fish cells, either alone or in combination with FGF-2 (Walsh et al., 2024). Similarly, Insulin-like growth factor-1 (IGF-1) is routinely added to fish cell culture media due to its crucial role in regulating vertebrate growth and development (Ndandala et al., 2022). Interleukin-13 (IL-13), a pleiotropic cytokine, is primarily studied in fish for its pro-inflammatory role in the immune response system, achieved by stimulating immune cell proliferation (Zou and Secombes, 2016). In mammals, it has been shown to induce proliferation in various epithelial cell types, such as esophageal (Marella et al., 2023) and conjunctival epithelial cells (Tukler Henriksson et al., 2015). In addition, it has been associated with tissue regeneration and maintenance of homeostasis (Roeb, 2023). Type II interferon (Interferon-gamma, IFN-γ) in fish is increasingly recognized for its role in regulating cell proliferation and developmental processes. In zebrafish embryos, IFN-γ has been implicated in the formation of hematopoietic stem cells. Moreover, it influences key developmental processes, such as the biliary system formation and germ cell proliferation in related fish species. These findings suggest that this cytokine may play a supportive role in maintaining and promoting the growth of larval-derived cell lines (Gan et al., 2020). Interleukin 15 (IL-15) is a pleiotropic cytokine with a broad tissue distribution, ranging from lymphoid organs to muscle tissues (Zhang and Su, 2023). It exhibited context-dependent functions including promotion of proliferation or hypertrophy (Budagian et al., 2006).

The cell line used here has already demonstrated its value as a model for climate change and toxicological studies (Grunow et al., 2011; Yebra-Pimentel et al., 2019; Grunow et al., 2021; Lutze et al., 2024) and its application is expected to expand further due to the dramatic conservation status of the sturgeon animal group and its relevance in both aquatic ecosystems and aquaculture production (Bronzi et al., 2011; Carrizo et al., 2017; Congiu et al., 2023). To promote this broader utilization, we aim to start the transition toward an animal-free cultivation system and to enhance its metabolic characterization, an essential critical aspect in toxicology that is often overlooked when adapting the cell line to new culture media. Mitochondrial health and activity, which are influenced not only by the nutrient composition of the culture medium but also represent key targets of chemicals and pollutants (Souders et al., 2018), are particularly important in this context. Therefore, metabolic changes induced by novel culture conditions could also alter cell responses to the toxins (Arodin Selenius et al., 2019). For this reason, we investigated the effects of serum reduction and supplementation with selected GFs and CKs on the AOXlar7y cell line proliferation and metabolism. This study serves as a foundation for designing an optimized, species-specific medium and marks a significant step forward in the establishment of a serum-free cultivation system for Atlantic sturgeon larval cell lines. Nevertheless, the tested GFs and CKs represent only a subset of variables necessary for designing a complete medium, and given that the experiments were conducted under short-term conditions, further systematic and long-term studies are needed to fully eliminate undefined and animal-origin ingredients in the AOXlar7y cells cultivation.

## 2 Materials and methods

### 2.1 Cell culture

The cell line AOXlar7y (Atlantic sturgeon; *Acipenser oxyrinchus*, larvae), obtained from the German Cell Bank for Wildlife (Fraunhofer EMB, Lübeck, Germany), was used (Grunow et al., 2011). The cells were cultivated at 25 °C in 150 cm^2^ flasks (TPP, Trasadingen, Switzerland) with a culture medium composed of Leibovitz’s-15 medium (L-15, Gibco, Darmstadt, Germany) supplemented with 10% FBS (Pan-Biotech, Adenbach, Germany) and 1% (v/v) penicillin/streptomycin (P/S; Gibco). Cells were passaged every 6 to 8 days at a ratio of 1:2 to 1:3. Passaging involved washing with 1× Dulbecco’s phosphate-buffered saline (DPBS; PanBioTech) and trypsinisation with 0.1% trypsin/EDTA solution (Gibco) for 3 min at 37 °C. A double amount of cell culture medium was added to stop the trypsin activity. The cell suspension was centrifuged for 5 min at 130 rcf, and the pelleted cells were seeded in new culture vessels. Three consecutive passages were used for each of the three experimental replicates in which cells were seeded in parallel in the 96-well RTCA E plate (OMNI Life Science GmbH and Co. KG, Bremen, Germany) for the cell impedance analysis and in the XFe96 culture microplates (Agilent; Santa Clara, CA, United States) for the mitochondrial stress test and for obtaining the phase-contrast pictures to assess morphology and cell numbers.

### 2.2 Media composition

Fifteen different media were prepared to evaluate the possibility of reducing serum and substituting it with GFs and CKs while cultivating AOXlar7y cells. The basic medium used for each condition was L-15 medium with 1% P/S. For the serum reduction groups, three media supplemented with 10% (as the control), 5% (mild reduction), and 2% (strong reduction) of FBS were used. To evaluate the effects of GF and CK addition, L-15 media containing 2% FBS were supplemented with 10 ng/mL or 50 ng/mL of the two tested GFs and four tested CKs (PeproTech^®^, now part of Thermo Fisher Scientific Inc.) (Table 1). Each GF and CK presented in this study was tested separately. The cultivation of the cells under serum-reduced conditions and GF and CK supplementation was not carried out consecutively over multiple passages, but was performed exclusively in the isolated experiments.

**TABLE 1:** Information about the growth factors (GFs) and cytokines (CKs) used. The name, type, category, and catalog number are listed.

Growth factor/Cytokines (10 and 50 ng/mL)		Extended name	Type	Category	Catalog#
Growth Factors	FGF-2	Fibroblast Growth Factor 2	Murine	Animal-Free manufactured	AF-450-33
	IGF-1	Insulin-like growth factor-1	Human	Animal-Free manufactured	AF-100-11
Cytokines	LIF	Leukemia Inhibitory Factor	Human	—	300-05
	IFN-γ	Interferon gamma	Human	Animal-Free manufactured	AF-300-02
	IL-13	Interleukin-13	Human	—	200-13
	IL-15	Interleukin 15	Murine	—	210-15

### 2.3 Cell morphology and cell density

To assess the impact of the GFs and CKs on cell morphology, AOXlar7y cells were cultivated in the experimental media and one phase contrast picture per well was taken on day six of cultivation with the Keyence microscope BZ-X800 (Keyence, Neu-Isenburg, Germany). The experiment was conducted in two independent runs (n = 2), each comprising biological replicates of n = 5 for the serum concentration groups and n = 6 for the GF and CK groups. Cell images were adjusted for brightness and contrast using Adobe Photoshop CC 2019 (Adobe Inc.). Automated cell count was performed using the software Olympus cellSens Dimension (Version 3.2, Olympus Corporation (today: Evident Co., Ltd.), Tokio, Japan) to assess the end-point cell density. A custom macro was created with the following steps: images were first opened and subjected to automatic shading correction (default settings), followed by the application of a smoothing filter (NxN, default settings). Subsequently, the Count & Measure module was initiated. For segmentation, a manual threshold value was set for each image to ensure consistent and accurate object detection. The minimum object size parameter was adjusted as necessary to exclude background noise and artifacts. When automatic segmentation proved inadequate, manual correction was performed, including the addition, deletion, or splitting of objects. After verification of segmentation accuracy, quantification data were exported to GraphPad Prism software, version 10.4.1 (GraphPad Software, Inc., La Jolla, CA, United States) for further analysis.

The images were calibrated using Fiji, a distribution of ImageJ (Schindelin et al., 2012), to determine the physical area they represented by relating pixel dimensions to a known scale. Specifically, a 100 μm scale bar corresponded to 92 pixels, yielding a pixel size of 1.087 μm per pixel. The total image area was calculated based on pixel calibration, resulting in 0.966 mm^2^ per image. This value was subsequently used to normalise the cell densities, expressed in cells per mm^2^.

### 2.4 xCELLigence real-time cell analyzer

Cell impedance (CI) detects modifications in the electrical resistance of gold microelectrodes (electric potential: 22 mV) caused by changes in cell number, cell size, cell adhesion and cell morphology. The more cells that attach and spread out on the electrodes, the greater the impedance and this is why the CI is increasing. The CI of the AOXlar7y cells was recorded every 15 min for 7 days using the xCELLigence RTCA SP instrument (Agilent; Santa Clara, CA, United States) to evaluate the effects of reducing serum concentration and adding GFs and CKs. 8,000 cells per well were seeded with 200 µL of 10% FBS L-15 medium and incubated at 25 °C in a 96-well RTCA E-plate (OMNI Life Science GmbH and Co. KG, Bremen, Germany) for cell attachment. The seeding density has been optimized to ensure the visualization of cell growth dynamics over multiple days. After 24 h, the medium was replaced with the experimental ones, and the CI was measured over 6 days (total experiment time was 7 days). The experiment was repeated three times, each time including technical replicates of n = 5 for the serum concentration groups and n = 6 for the GF and CK groups. Curves, slopes, and doubling times were obtained using RTCA Software 1.2.1. The slope and the doubling time were determined from the linear phase of each curve (hours 50–100). Maximal CI was identified as the highest value reached during the 6 days of observation. Maximal CI and slope values were then normalized to the 10% FBS control on the same plate by calculating the ratio of each treatment’s maximal CI to that of the 10% FBS condition. This normalization minimized inter-plate variability, enabling a more direct comparison of the experimental conditions across experiments.

### 2.5 Seahorse mitochondrial stress test

To assess mitochondrial and metabolic modifications under serum reduced medium and with the GFs and CKs supplementation, AOXlar7y cells were seeded in XF96 culture microplates (Agilent; Santa Clara, CA, United States) at a concentration of 8,000 cells per well with 200 µL of 10% FBS medium and incubated at 25 °C. After 24 h, the medium was substituted with the experimental ones. Six days after the medium substitution, the Mito Stress Test (#103015-100, Agilent) was performed to measure mitochondrial respiration using Seahorse Technology (Agilent). For this purpose, the incubated cells were washed twice with XF Cell Mito Stress Test Assay medium containing glucose (1 mM), pyruvate (1 mM), and L-glutamine (2 mM), and then incubated within this media at 25 °C for 1 h.

After preparing and loading the compounds from the XF Cell Mito Stress Test Kit (Agilent Technologies) into the designated cartridge ports, the cartridge was inserted into the XFe96 Seahorse Analyzer, followed by the placement of the cell culture plate. Following an initial equilibration phase, a series of measurement cycles was carried out. Three recording cycles were performed first to assess basal oxygen consumption rate (OCR–basal respiration). Subsequently, oligomycin (final concentration 2 µM) was added to inhibit ATP synthase, allowing the calculation of the portion of oxygen consumption linked to ATP production and proton leak. After three additional measurement cycles, FCCP (carbonyl cyanide-4-(trifluoromethoxy) phenylhydrazone; final concentration 1 µM) was injected to uncouple oxidative phosphorylation, thereby revealing the maximal mitochondrial respiration capacity (R_max_). Finally, to quantify the non-mitochondrial oxygen consumption, a mixture containing rotenone and antimycin A (each at 0.5 μM), which inhibit respiratory complexes I and III, was added, followed by another three measurement cycles.

The same experiment was performed twice, each time including biological replicates of n = 5 for the serum concentration groups and n = 6 for the GF and CK groups. All data were used to calculate basal and maximal respiration, ATPase-linked ATP production, proton leak and spare respiratory capacity using Agilent’s software (WAVE, Toronto, ON, United States, version 2.6.1).

### 2.6 Energetic phenotype analysis

Measurements regarding the extracellular acidification rate (ECAR) and OCR of the Mito Stress Test from the two independent experimental replicates with each experimental trial having 5-6 biological replicates were further used to evaluate the relative bioenergetic phenotype of the cells by using the analysis pattern ‘Cell Energy Phenotype Test Report Generator’ provided by the WAVE program. Therefore, ECAR (x-axis) and OCR (y-axis) values of baseline and uncoupled respiration (“stressed”) conditions were plotted in a diagram. Low ECAR and OCR values indicated a quiescent state. Low ECAR and high OCR values indicated the utilization of predominantly mitochondrial respiration, while low OCR and high ECAR values displayed predominantly glycolytic utilization. High ECAR and OCR values implied the utilization of both metabolic pathways.

### 2.7 Protein measurement

To reduce the influence of the variation in cellular number across the tested conditions during 7-day cultivation, the protein contents per well were quantified after the Seahorse experiments, following the protocol described by Wanka et al. (2018). The previously frozen Seahorse plates were thawed, and the cells were lysed in 12 μL of RIPA buffer containing 33.3 mmol/L Tris, 3.33 mmol/L EDTA, 100 mmol/L NaCl, 6.67 mmol/L K2HPO4, 6.67% glycerol, 0.67% TritonX-100, 1 mmol/L NaVO4, 20 mmol/L NaF, 0.1 mmol/L PMSF, 20 mmol/L 2-phosphoglycerat, plus a protease inhibitor cocktail (Roche Diagnostics). 50 μL of the Bradford reagent (Roth, Karlsruhe, Germany) was added to 50 μL of the 1:10 diluted sample to measure the protein content according to the manufacturer’s instructions (Roth, Karlsruhe, Germany).

### 2.8 Statistical analysis

Statistical analyses were performed using GraphPad Prism software, version 10.4.1 (GraphPad Software, Inc., La Jolla, CA, United States). An ordinary one-way ANOVA with Dunnett correction was applied for the statistical testing of the results obtained from the cell count, where biological replicates were treated as independent values. The ordinary one-way ANOVA with Dunnett correction and Kruskal–Wallis test with Dunn’s post-hoc correction were applied to the statistical testing of the results obtained from the xCELLigence Real-Time Cell Analyzer. Technical replicates were first averaged and the statistical test was based only on experimental/biological replicates. For the Seahorse Mitochondrial Stress Test and the Energetic Phenotype Analysis, statistical analyses were performed by pooling the biological replicates of the two experiments together, treating them as independent data points. Prior to analysis, data were background-corrected and normalized to individual protein content. The statistical difference was intended as p* < 0.05, ** ≤ 0.01, *** ≤ 0.001, **** ≤ 0.0001. Data are presented as mean ± SD.

## 3 Results

In this study, the impact of the serum-reduced media and the supplementation of two concentrations of six different GFs and CKs were investigated on the morphology, cell growth, and oxygen consumption in basal and uncoupled respiration conditions of the AOXlar7y cell line. Cells were exposed to the experimental media for 6 days to assess the resilience of sturgeon cells under serum deprivation and examine the direct effects of GFs and CKs.

### 3.1 Cell morphology and cell density

**FIGURE 1:**
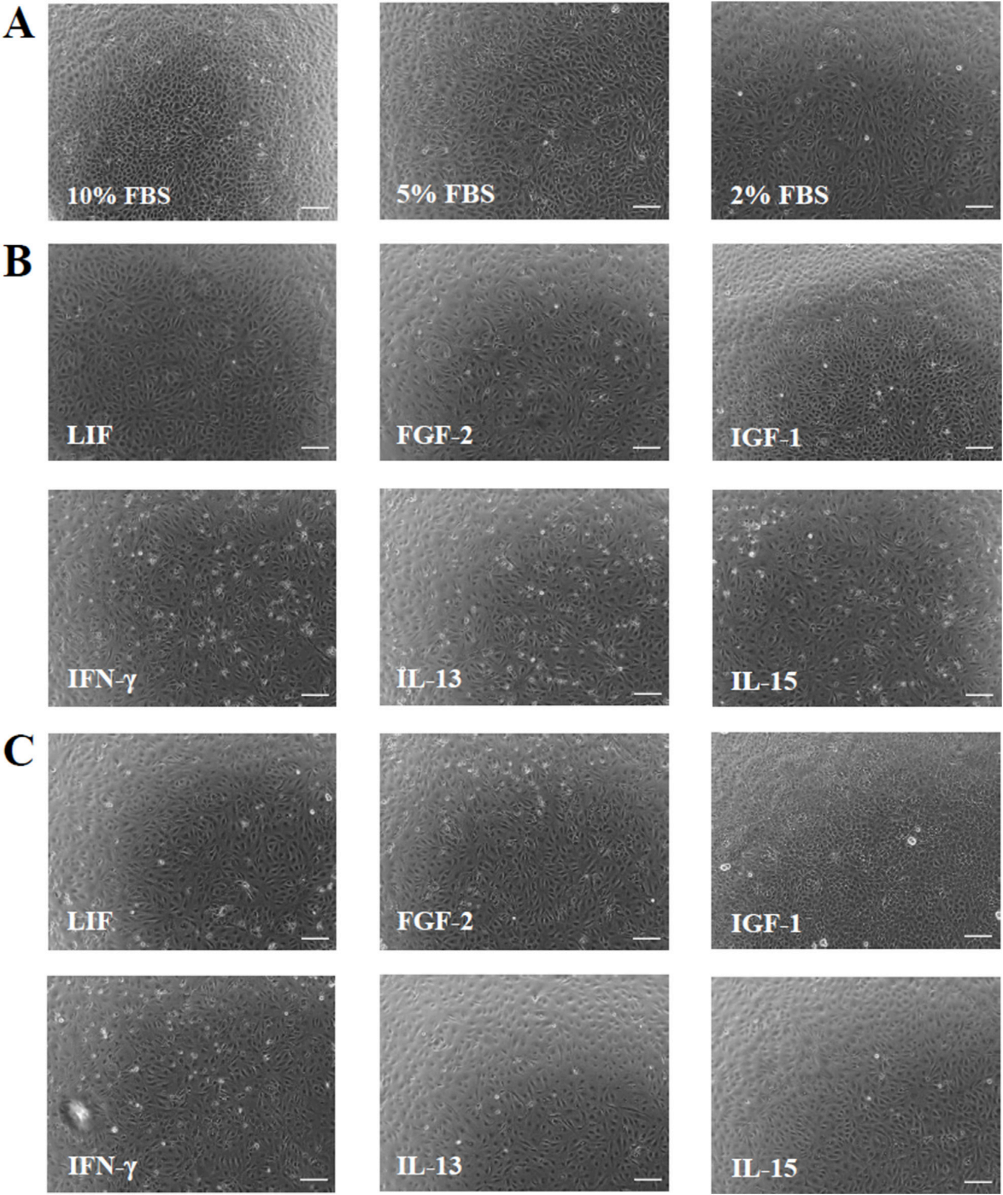
Phase contrast cell images showing cell morphology and density of AOXlar7y cells after 6 days of cultivation. **(A)** Three FBS concentrations were tested (10% as the standard, 5% as medium reduction, and 2% as strong reduction). Regarding the GF and CK supplementation, media with strong serum reduction (2% FBS) were mixed with **(B)** 10 ng/mL and **(C)** 50 ng/mL of each GF/CK: Leukemia Inhibitory Factor (LIF), Fibroblast Growth Factor 2 (FGF-2), Insulin-like Growth Factor 1 (IGF-1), Interferon-gamma (IFN-γ), Interleukin-13 (IL-13), and Interleukin-15 (IL-15). Scale bar: 100 μm.

**FIGURE 2:**
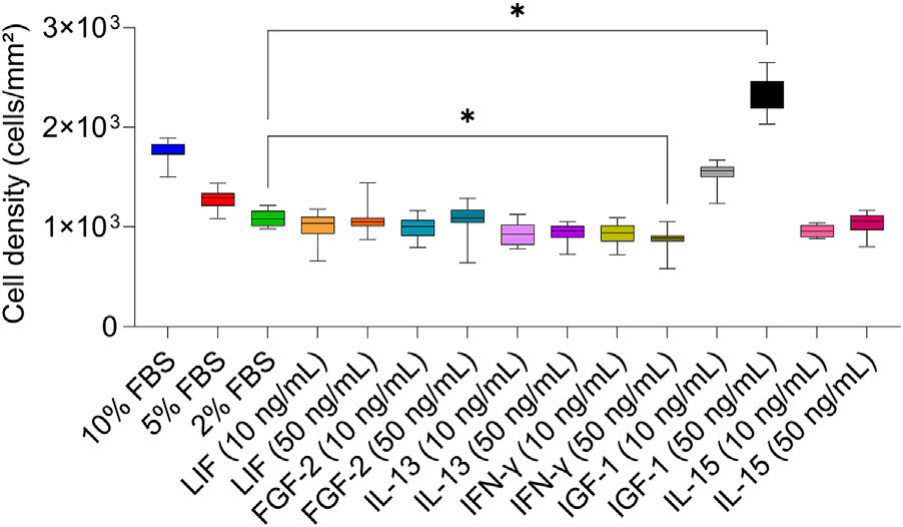
Cell density of AOXlar7y cell line obtained by phase-contrast microscope pictures in response to serum concentration and GFs and CKs supplementation. Experimental trials: three FBS concentrations were tested (10% as the standard - blue, 5% as medium reduction - red, and 2% as strong reduction - bright green). Media with strong serum reduction (2% FBS) were supplemented with 10 ng/mL (lighter color) and 50 ng/mL (darker color) of each GF/CK: Leukemia Inhibitory Factor (LIF) - orange, Fibroblast Growth Factor 2 (FGF-2) – blue-green, Interleukin-13 (IL-13) - purple, Interferon-gamma (IFN-γ) – yellow-green, Insulin-like Growth Factor 1 (IGF-1) – grey, and Interleukin-15 (IL-15) - pink. The experiment was repeated twice, each time including biological replicates of n = 5 for the serum concentration groups and n = 6 for the GF and CK groups. Data are presented as mean ± SD of all biological replicates. Ordinary One-Way ANOVA with Dunnett’s post-correction was applied to compare each experimental group to the 2% FBS group. The statistical difference was intended as p*<0.05, **≤0.01, ***≤0.001, ****≤0.0001.

The phase-contrast images taken after 6 days of cultivation with serum-reduced media and with the two concentrations of the GFs and CKs showed no significant modifications in cell morphology compared to 10% FBS (Figures 1A–C). Overall, cells with lower cell densities exhibited flatter and larger shapes compared to standard conditions. Cell numbers retrieved by the phase-contrast images confirmed the presence of different cell densities (Figure 2). A concentration-dependent reduction in cell number was observed with the two serum-reduced conditions (5% and 2% FBS). The cells treated with GFs and CKs, which were also supplemented with 2% serum, showed a comparable density to the 2% FBS group. However, the cells treated with IGF-1 exhibited an increase in cell density, with the 50 ng/mL concentration being higher than the 10% FBS group.

### 3.2 Cell impedance analysis

**FIGURE 3:**
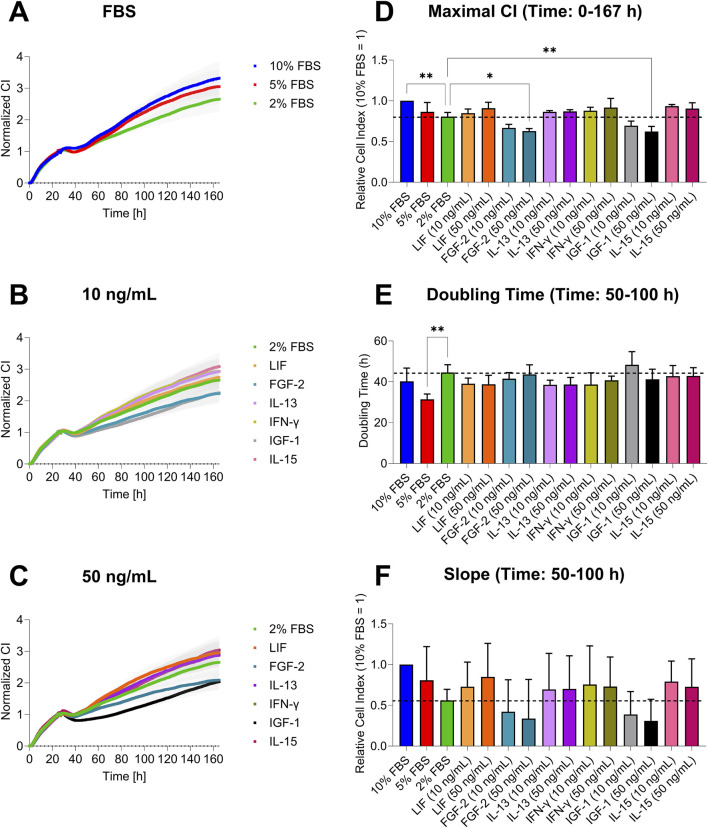
Cell growth analysis of AOXlar7y cell line by impedance measurement in response to serum concentration reduction and GFs and CKs supplementation. **(A–C)** Growth curves show the CI over 7 days (mean ± SEM). **(D)** Maximal CI represents the highest value reached expressed in ratios (10% FBS ratio = 1). **(E)** Doubling time and **(F)** growth slope expressed in ratio (10% FBS ratio = 1) were calculated for the hourly range from 50 to 100. Experimental trials: three FBS concentrations were tested (10% as the standard - blue, 5% as medium reduction - red, and 2% as strong reduction - bright green). Media with strong serum reduction (2% FBS) were supplemented with 10 ng/mL (lighter color) and 50 ng/mL (darker color) of each GF/CK: Leukemia Inhibitory Factor (LIF) - orange, Fibroblast Growth Factor 2 (FGF-2) – blue-green, Interleukin-13 (IL-13) - purple, Interferon-gamma (IFN-γ) – yellow-green, Insulin-like Growth Factor 1 (IGF-1) – grey, and Interleukin-15 (IL-15) - pink. The experiment was repeated thrice, each time including replicates of n = 5 for the serum concentration groups and n = 6 for the GF and CK groups. Data are presented as mean ± SD of the biological replicates. Ordinary One-Way ANOVA **(D)** with Dunnett’s post-correction and Kruskal–Wallis test with Dunn’s post-correction **(E and F)** was applied for group comparisons. The statistical difference was intended as p*<0.05, **≤0.01, ***≤0.001, ****≤0.0001.

The graphs showed distinct patterns in cell growth across the conditions (Figure 3). Cells with standard serum (10%) had the highest CI, while mild serum reduction (5%) caused a slight decrease, and strong reduction (2%) led to a more linear growth (Figure 3A). Cells treated with 10 ng/mL of GFs and CKs had a similar pattern to the 2% FBS group, but CI varied by GF/CK type: FGF-2 and IGF-1 showed a decreased CI, while LIF, IFN-γ, IL-13, and IL-15 increased cell growth, with IL-15 showing the highest impedance (Figure 3B). At 50 ng/mL, FGF-2 and IGF-1 had the lowest signals, with IGF-1 exhibiting a prolonged lag phase. LIF’s CI increased at the higher concentration, approaching the 5% serum level (Figure 3C). Differences between experimental groups became apparent 30–40 h after the medium exchange.

Overall, no significant differences were found between all experimental groups for the maximal CI and the growth slope (Figures 3D,E). The maximal CI shows that the highest impedance over the cultivation was reached by the standard condition, followed by 5% FBS, LIF (50 ng/mL), IL-13, IFN-γ, and IL-15 (all with both concentrations). The lowest values are seen in FGF-2- and IGF-1-supplemented media (Figure 3D). Analysis of the doubling time revealed a duration of 40–50 h for most conditions, with a slight overall reduction in doubling time compared to the 2% FBS group. However, this trend was not observed for FGF-2 (50 ng/mL), IFN-γ (50 ng/mL), and IGF-1 (10 ng/mL) conditions. Statistically significant differences were found between the 5% FBS and 2% FBS groups (Figure 3E). The growth slope was similar for the 5% and 10% FBS groups. LIF, IL-13, IFN-γ, and IL-15 had higher slopes than the 2% FBS group, with LIF (50 ng/mL) showing the highest increase. FGF-2 and IGF-1 displayed the lowest slope values (Figure 3F).

### 3.3 Mitochondrial stress test

**FIGURE 4:**
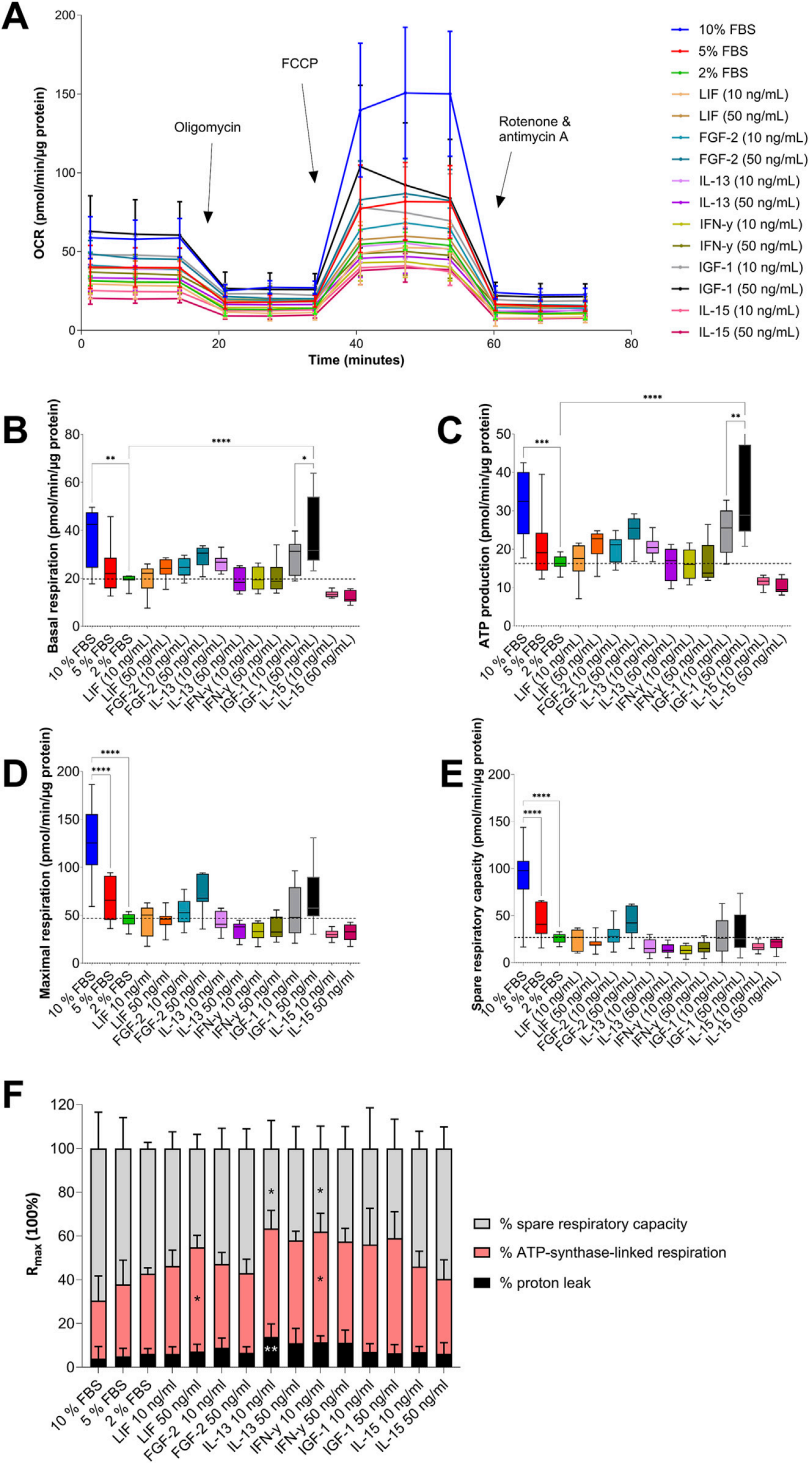
Analysis of mitochondrial respiration in AOXlar7y cells in response to serum concentration reduction and GF/CK supplementation. **(A)** The OCR (Oxygen Consumption Rate) was measured with the Mito Stress test under basal conditions and after administering oligomycin, FCCP, and rotenone/antimycin A to modulate mitochondrial respiratory chain proteins. **(B)** Basal respiration, **(C)** ATP production, **(D)** maximal respiration, and **(E)** spare respiratory capacity were calculated with normalised data obtained. The percentage of spare respiratory capacity, ATP synthase-linked respiration, and proton leak from maximal respiration is shown in **(F)**. Experimental trials: three FBS concentrations were tested (10% as the standard - blue, 5% as medium reduction - red, and 2% as strong reduction - bright green). Media with strong serum reduction (2% FBS) were supplemented with 10 ng/mL (lighter color) and 50 ng/mL (darker color) of each GF/CK: Leukemia Inhibitory Factor (LIF) - orange, Fibroblast Growth Factor 2 (FGF-2) – blue-green, Interleukin-13 (IL-13) - purple, Interferon-gamma (IFN-γ) – yellow-green, Insulin-like Growth Factor 1 (IGF-1) – grey, and Interleukin-15 (IL-15) - pink. The experiment was repeated twice, each time including biological replicates of n = 5 for the serum concentration groups and n = 6 for the GF and CK groups. Data are presented as mean ± SD of all biological replicates. Ordinary One-Way ANOVA **(B–F)** was applied for group comparisons where the statistical difference was intended as p*<0.05, **≤0.01, ***≤0.001, ****≤0.0001.

Analysis of mitochondrial respiration revealed modulation by supplementation of different GFs and CKs (Figure 4). None of the GFs and CKs used were able to neutralize the reduction in OCR of AOXlar7y cells cultivated with 2% FBS compared to those cultivated with 10% FBS (Figure 4A). However, basal respiration (Figure 4B) and ATP synthase-linked respiration (Figure 4C) of AOXlar7y cells in 2% FBS supplemented with 50 ng/mL IGF-1 corresponded to those of the cells cultivated in 10% FBS. Both basal respiration and ATP-linked respiration differed significantly between cells in 2% FBS media without any addition and cells cultivated with 10 ng/mL IGF-1. Other supplements, such as 50 ng/mL LIF, 10 ng/mL or 50 ng/mL FGF-2, and 10 ng/mL IL-13, increased the basal respiration and ATP-linked OCR of the cells slightly but not significantly, comparable to the basal respiration of cells incubated with 5% FBS. In contrast, IL-15 decreased basal respiration and ATP-linked OCR at both concentrations compared to 2% FBS-treated cells.

The maximal respiration capacity (Figure 4D) and spare respiratory capacity (Figure 4E) of cells incubated with FBS decreased dose-dependently. However, the maximal respiration capacity and spare respiratory capacity of cells treated with 10% FBS could not be achieved by any GF or CK supplementation to 2% FBS media. Only the addition of IGF-1 and FGF-2 to the medium at high concentrations increased the respiration to the level of cells cultured with 5% FBS. All four CKs, LIF, IFN-γ, IL-13, and IL-15, markedly reduced spare respiratory capacity and, in some cases, maximal respiration compared to the 2% FBS medium control.

Interestingly, the percentage composition of the O_2_-consuming sub-processes changed in response to the addition of CKs (Figure 4F). Adding 50 ng/mL LIF or 10 ng/mL IFN-γ significantly increased the proportion of ATP synthase-linked respiration, while 10 ng/mL IFN-γ or IL-13 significantly reduced the spare respiratory capacity. In addition, 10 ng/mL IL-13 increased the proton leak compared to 2% FBS-containing medium. Nevertheless, these CK-related changes also differed significantly from the percentages of maximal respiration of cells incubated in 10% FBS.

**FIGURE 5:**
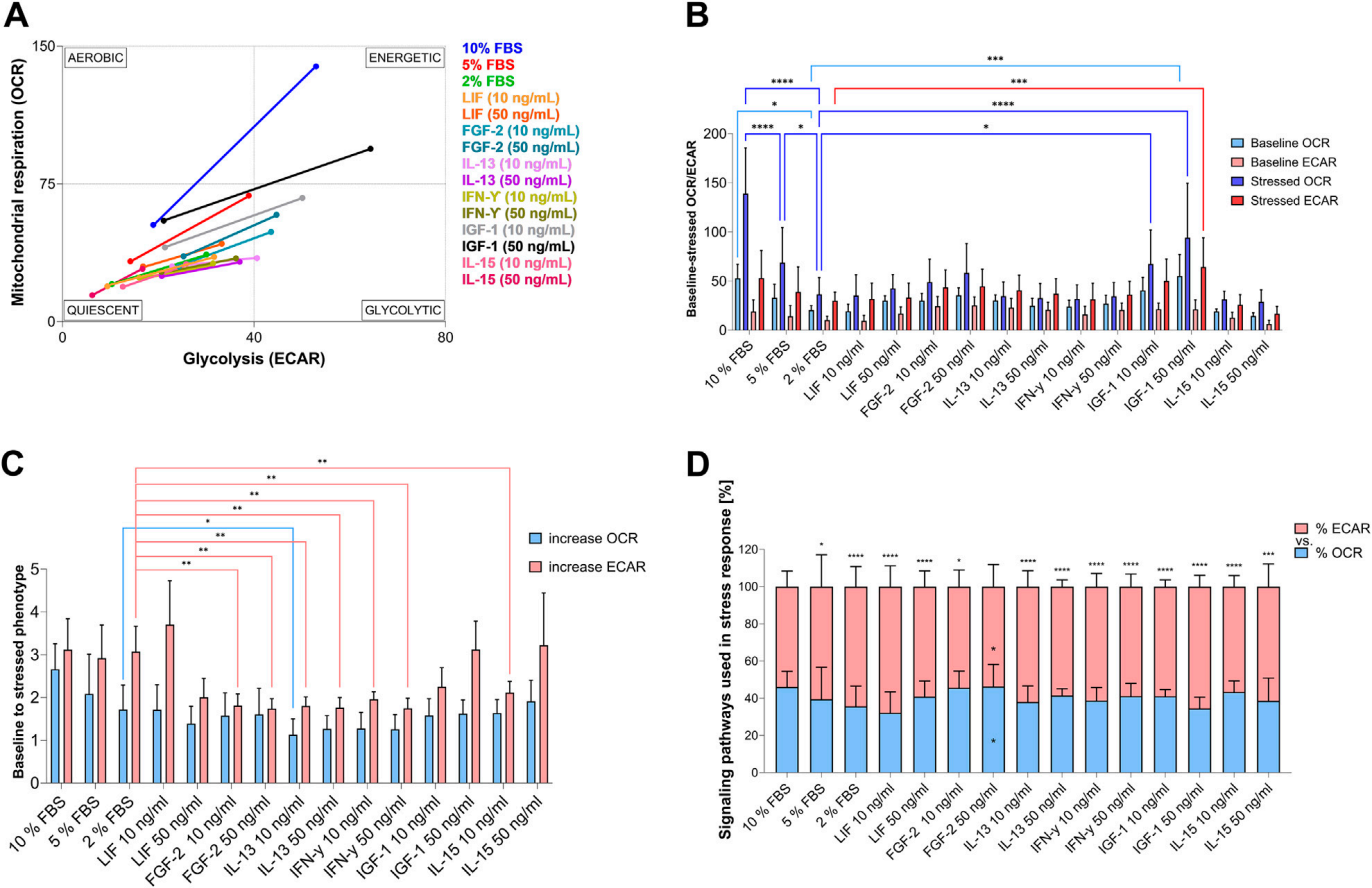
Relative bioenergetic phenotypes of AOXlar7y cells cultivated in different serum concentrations and GFs and CKs supplementations. **(A)** Analysis of energy phenotype transition from baseline (lower value) to stressed conditions (higher value) was determined by measuring extracellular acidification rate (ECAR) and oxygen-consumption rate (OCR) using the Mito Stress Test Assay. **(B)** The corresponding baseline and stressed OCR and ECAR values, and **(C)** the increase between baseline and stressed conditions are presented as bar charts. **(D)** The proportion of oxidative and glycolytic signaling pathways in response to stress. Experimental trials: three FBS concentrations were tested (10% as the standard - blue, 5% as medium reduction - red, and 2% as strong reduction - bright green). Media with strong serum reduction (2% FBS) were supplemented with 10 ng/mL (lighter color) and 50 ng/mL (darker color) of each GF/CK: Leukemia Inhibitory Factor (LIF) - orange, Fibroblast Growth Factor 2 (FGF-2) – blue-green, Interleukin-13 (IL-13) - purple, Interferon-gamma (IFN-γ) – yellow-green, Insulin-like Growth Factor 1 (IGF-1) – grey, and Interleukin-15 (IL-15) – pink (colors were applied in **A**). The experiment was repeated twice, each time including biological replicates of n = 5 for the serum concentration groups and n = 6 for the GF and CK groups. Data are presented as mean ± SD of all biological replicates. A Two-Way ANOVA test with Tukey’s multiple comparisons test **(C)** and a Mixed-Effects analysis with Tukey’s multiple comparisons test **(B and D)** were applied. The statistical difference was intended as p*<0.05, **≤0.01, ***≤0.001, ****≤0.0001. Asterisks on top of the bar chart **(D)** indicate the statistical difference between the increase in OCR and the increase in ECAR per each condition, while internal asterisks indicate the statistical differences between the tested condition with 2% FBS.

Simultaneous observation of mitochondrial respiration and glycolysis under baseline and stress conditions allows the assessment of the relative bioenergetic phenotype of control and treated cells (Figure 5). In response to the mitochondrial treatment (Mito Stress Test), AOXlar7y cells cultivated in 10% FBS showed a high energetic response, indicating that they utilize glycolytic and oxidative pathways for energy production. In comparison, reducing FBS in the media decreased the energetic phenotype under baseline and the use of the Mito Stress Test components (Figures 5A,C). Under baseline conditions, the energetic phenotype shifted to a more quiescent one and toward glycolytic utilization in response to stress (Figures 5A,C). In turn, adding different GFs or CKs to 2% FBS media slightly increased baseline ECAR, except for LIF (10 ng/mL) and IL-15, and baseline OCR exclusively for LIF (50 ng/mL), IL-13 (10 ng/mL), FGF-2, and IGF-1 (significantly at 50 ng/mL). However, cells treated with 50 ng/mL of IGF-1 exhibited a comparable basal phenotype to the 10% FBS control (Figures 5A,C) and an equivalent increase in glycolytic utilization in response to the induced mitochondrial stress (Figures 5A,B) but with a lower dependence on oxidative pathways (Figure 5D). Interestingly, besides 50 ng/mL IGF-1, comparable ECAR increases, similar to those in 10% FBS-cultivated cells, in response to stress were also observed with 5% and 2% FBS, as well as 50 ng/mL IL-15 (Figure 5D). Ten ng/mL LIF in 2% FBS medium led to an even higher ECAR increase than the addition of 10% FBS. However, the baseline metabolic phenotype (Figure 5C) and OCR increased in response to the induced uncoupled mitochondrial respiration of cells cultivated with lower FBS concentrations and 50 ng/mL IL-15 (Figure 5D) were not equivalent to that of the 10% FBS group. In general, neither GF nor CK supplementation could increase the oxidative utilization of cells in response to induced mitochondrial stress, as observed in the 10% FBS group (Figure 5C).

Notably, supplementation with 50 ng/mL FGF-2 showed the same percentage increase in glycolytic and oxidative metabolic pathways under stress conditions compared to 10% FBS (Figure 5D). Both pathways were equally favored, whereas in all other GFs supplementation, metabolic stress induced a greater increase in glycolytic signaling pathways. Thus, the percentage distribution of increases in 50 ng/mL FGF-2-treated cells differed significantly from those cultured with 2% FBS.

## 4 Discussion

Fish cell lines derived from larvae are relatively rare (Bairoch, 2018), and toxicity testing still largely relies on live fish larvae to capture systemic responses from whole organism (Xiong et al., 2022). To date, AOXlar7y is the only larval cell line available for the *Acipenser* genus listed in the Cellosaurus database (Bairoch, 2018). Sturgeons have recently been declared the most endangered animal group in the world, according to the latest assessment of their conservation status by the International Union for Conservation of Nature (IUCN) (Congiu et al., 2023). Understanding animal physiology is therefore crucial for species conservation and environmental policy implementation, especially for species and ecosystems under increasing anthropogenic pressure (Dubuc et al., 2025). Fish represent the most diverse vertebrate group, with approximately 36,000 species (FishBase, 2025). Capturing species-specific physiological responses is essential for a comprehensive understanding of how environmental contaminants affect aquatic ecosystems’ health, particularly in the context of interacting stressors such as rising temperatures and lower dissolved oxygen driven by climate change. The temperature increase proved to have an influence on the toxicity of the chemicals tested in the AOXlar7y cell line. Moreover, the observed toxicology dynamics appeared to be species-specific when compared to the responses of a fish cell line derived from another species (Grunow et al., 2021).

Previous studies have demonstrated the potential of whole larvae fish cell lines to span various applications, ranging from climate change and toxicology studies to the development of specialized systems, such as *in vitro* contracting cardiomyogenic models (Hodgson et al., 2018; Noguera et al., 2021; Friesen et al., 2025). By improving the characterization, optimization, and standardization of fish larvae cell lines, sectors such as toxicology, biodiversity conservation, sustainable aquaculture, and further biomedical and biotechnological applications can benefit from this *in vitro* model (Mushtaq et al., 2025). The AOXlar7y cell line is currently cultivated in standard serum-supplemented culture medium, and to our knowledge, no species-specific, validated, chemically defined, or animal-free media are available for sturgeon cell cultures. Therefore, in this study, we explored the effects of serum reduction and the addition of two concentrations of six GFs and CKs on cell morphology, cell growth, and metabolism of the AOXlar7y cell line. The aim was to initiate the transition toward a fully FBS-free cultivation system.

### 4.1 Serum reduction

Serum reduction can influence cell morphology, growth, and metabolism depending on the extent of reduction and cell type (Pirkmajer and Chibalin, 2011; Jang et al., 2022; Giannasi et al., 2023). This aspect requires attention while transitioning to a serum-free medium. As a matter of fact, toxicological tests may be influenced by the use of serum, with results varying depending on serum concentration, underlying the need to explore the correlation between cell responses and serum availability (Clift et al., 2010; Zhang et al., 2020; Khare et al., 2022a; Khare et al., 2022b; Wei et al., 2022; Santos et al., 2023). Fish cell lines often undergo major morphological and cell number changes when cultured under serum-restricted conditions. For example, RTgutGC intestinal cells from rainbow trout exhibit a more adherent, larger, flatter, and polygonal morphology after 7 days in serum-free media (Pumputis et al., 2018). In contrast, ZF4 embryonic cells from zebrafish exhibit a shorter and rounder shape within just 2 days, accompanied by impaired adhesion and decreased cell viability (Fan et al., 2020). Zebrafish embryonic stem cells (ZEMS2) show an unchanged morphology but reduced cell density after 3 days in serum-reduced culture (Batish et al., 2022). Our study demonstrated the general morphological stability of AOXlar7y cells cultured under reduced serum conditions for 6 days across all tested treatments. However, a decrease in cell number was observed in the 5% FBS and 2% FBS groups, except for the IGF-1-treated cells, where both tested concentrations of IGF-1 appeared to promote cell proliferation and effectively compensated the serum reduction. At lower cell densities, the cells appeared flatter and larger. Nevertheless, all groups reached cell confluence after 7 days in culture.

For further characterization of the cells, xCELLigence analysis was performed to determine variations in cell impedance in response to cell number, morphology, and adherence, as serum reduction generally decreases proliferation and induces cell adherence impairment and apoptosis. Stronger cell adhesion and a larger cell size lead to a more stable and higher CI (Kho et al., 2015). In AOXlar7y cells, serum reliance for the maintenance of sustained cell growth over a longer period was confirmed. However, 5% FBS elicited similar results in terms of performance compared to the standard serum concentration, which corresponds with the cell density observed in the phase contrast images. While cells cultivated with 2% FBS exhibited a more marked decrease in cell growth, this serum concentration was enough to keep the cells alive and active. A proportional decrease in CI with serum reduction was expected, however, these differences were less pronounced than anticipated, reflecting the potential of this cell line to adapt to a serum-free medium. However, long-term cultivation and serum depletion could have provided a more complete overview of AOXlar7y cell behavior. Metabolically, serum reduction was associated with a marked decline in basal and maximal respiration, spare respiratory capacity, and ATP-linked oxygen consumption, exhibiting a concentration-dependent shift toward a more quiescent state. A similar pattern was observed in human adipose-derived stem/stromal cells (ASCs), where 72 h of serum starvation led to a decline in basal, maximal and ATP-linked oxygen consumption (Giannasi et al., 2023). In zebrafish embryonic cells, the mitochondrial content appeared reduced after 12 h of serum deprivation (Fan et al., 2022), indicating the importance of specific serum-derived components in sustaining mitochondrial function and oxidative metabolism. Except for cancer cells, the nutrient uptake from the extracellular environment is mediated by growth factor signaling (Thompson and Bielska, 2019). The end-point reduced metabolism could be explained by the reduced amount of growth factors from the restricted FBS, combined with other potential elements from the serum. However, since major cell functions did not appear to be compromised, the observed reduced metabolic activity and proliferation in serum-reduced media does not necessarily limit the applicability of this *in vitro* model.

While supplementation with 10% FBS is still common in (mammalian) cell culture media due to its effect on increasing cell proliferation, it lacks species-specific and cell-type-specific optimization (Weber et al., 2025). Replacing 10% FBS with a defined formulation requires identifying the key factors that support the physiological characteristics of fish larvae *in vitro*—highlighting the need for targeted studies.

### 4.2 Supplementation with cytokines

Cytokines are an important component of serum, and all the major cytokine families have been described in fish, suggesting that the main aspects of immune responses are generally conserved and ancient in vertebrates (Zou and Secombes, 2016; Lee et al., 2022a). Cytokines act as signaling molecules regulating cell survival, proliferation, differentiation, and function (Schirmer and Neumann, 2019). They are generally studied for their crucial role in the immune system, but they can also influence the health, metabolism, and proliferation of various non-immune cells (Liu et al., 2023; Santos et al., 2023). In this current work, we tested four pleiotropic cytokines - LIF, IFNγ, IL-13, and IL-15 - involved in immune responses, stress responses, and cell survival in both mammals and fish.

Leukemia Inhibitory Factor (LIF), belonging to the Interleukin-6 family, is generally added in embryo cell cultures of both mammals and fish because it promotes self-renewal of stem cells, especially embryonic stem cells and neuronal stem cells (He et al., 2006; Hanington et al., 2008; Nicola and Babon, 2015; Wang et al., 2017). However, the effects are context-dependent. The addition of LIF and Glial Cell Line-Derived Neurotrophic Factor (GDNF) to the culture medium of an *Acipenser ruthenus* germ cell culture significantly increased mitotic activity compared to cultures without this (Xie et al., 2019). In contrast, LIF supplementation in embryonic stem cells from *Sciaenops ocellatus* elicited limited effects (Walsh et al., 2024). In the *Acipenser oxyrhinchus* cell line AOXlar7y used here, a concentration of 10 ng/mL LIF was insufficient to induce notable higher proliferation. In comparison, 50 ng/mL slightly increased cell growth, basal metabolism, and ATP-synthase-linked respiration, with the drawback of reducing the spare respiratory capacity. Based on current knowledge, the limited increase in cell proliferation suggests that this CK is not an ideal candidate for serum-free medium formulation. However, it may become more relevant if future studies using different concentrations demonstrate a clearer proliferation-supportive effect.

Interleukin-13 (IL-13) and Interferon gamma (IFN-γ) are immune-modulating cytokines known to influence and promote the proliferation of various cell types, including immune cells, murine mammary epithelial cells, and human bronchial epithelial cells *in vitro* (Asao and Fu, 2000; Gomez and Reich, 2003; Booth et al., 2007; Tukler Henriksson et al., 2015; Contreras-Panta et al., 2024). In AOXlar7y cells, IL-13 induced a slight increase in cell impedance without any concentration-dependent effect. Notably, 10 ng/mL of IL-13 resulted in a higher increase in basal respiration compared to 50 ng/mL. In a pre-print, Jackson et al. reported that in human patients with eosinophilic esophagitis, mitochondrial mass was increased in the esophageal epithelium in response to IL-13 (Jackson et al., 2025). In addition, this cytokine has been shown to enhance mitochondrial oxygen consumption in the murine muscle cell line C2C12 and is associated *in vivo* with endurance exercise (Webb and Tait Wojno, 2020). Interestingly, IL-13 markedly increased proton leak, suggesting overall a protective mechanism against the overproduction of Reactive Oxygen Species (ROS), particularly during inflammation (Nanayakkara et al., 2019). Bioenergetic analysis revealed a shift toward a less energetic, more glycolytic state, supporting the hypothesis that IL-13 may protect AOXlar7y cells from ROS-induced damage (Brand, 1997; Soto-Heredero et al., 2020). However, its limited proliferative effect indicates that IL-13 is not suitable as a supplement in serum-free medium formulation for AOXlar7y cells at the tested conditions. Similarly, IFN-γ showed limited ability to promote AOXlar7y cell proliferation, with better outcomes observed at lower concentrations. Like IL-13, IFN-γ increased proton leak and shifted the cells' metabolism toward a more glycolytic state compared to 2% FBS, potentially enhancing cells’ protection from ROS damage. Despite these metabolic adaptations, IFN-γ also appears unsuitable as a substitute for cultivating AOXlar7y cells.

Interleukin 15 (IL-15) is known to regulate and promote the proliferation of immune cells and other cell types, such as adult murine neural stem cells; however, its functions in fish cells are currently underexplored (Budagian et al., 2006; Gómez-Nicola et al., 2011). In AOXlar7y cells, IL-15 induced a slight increase in cell growth, promoted basal metabolism suppression, and induced the most quiescent phenotype at the higher concentration of all tested conditions. A previous study reported that continuous administration of IL-15 to human Natural Killer cells resulted in cell exhaustion (Felices et al., 2018). R_max_ was reduced in AOXlar cells cultivated with IL-15 compared to the 2% FBS control. This suggests impaired mitochondrial flexibility and a limited ability to meet increased energy demands. The shift may indicate a metabolic reprogramming toward glycolysis, consistent with IL-15’s role in activating immune cells. The reduced oxidative capacity is likely unfavorable for robust growth, making IL-15 an unsuitable mitogenic supplement for serum-free culture of AOXlar7y cells at the tested conditions. Nevertheless, IL-15 stimulated the oxidative metabolism of muscle cells, resulting in a marked increase in mitochondrial density (O'Connell and Pistilli, 2015). Therefore, the impact of IL-15 on cellular metabolism could be context and cell-type-dependent.

### 4.3 Supplementation with growth factors

Growth factors are essential serum supplements, supporting cell proliferation, survival, and metabolic activity. They help maintain physiological signaling pathways in serum-free or reduced-serum culture conditions. The mitogenic GFs, specifically, stimulate cell division by activating signaling pathways involved in the cell cycle. Therefore, FGF-2 and IGF-1, also tested in this study on AOXlar7y cells, are the main cost drivers in serum-free media formulation for large-scale applications like cellular agriculture. They account for more than 95% of the total estimated costs, making the selection of GFs of major relevance (Venkatesan et al., 2022). Moreover, currently commercially available animal-free GFs and CKs tend to be more expensive, despite their costs gradually aligning with the average price. They are generally validated only for their functional bioactivity, while non-animal-free counterparts are still more affordable and undergo a broader validation, including for their use in immunoassays. This makes it difficult for researchers to only rely on animal-free ingredients. As such, only three of the six recombinant proteins tested in the present study were of animal-free origin (Table 1).

The Fibroblast Growth Factor-basic (FGF-2) has a widely recognized mitogenic activity and is generally added to culture media of both mammals and fish cells for its ability to promote the proliferation of various cell types like human ear chondrocytes and embryonic cells from the fish red drum (*S. ocellatus*) (Mandl et al., 2004; Walsh et al., 2024). In AOXlar7y cells, FGF-2 induced discordant effects, characterized by a decrease in cell impedance with a simultaneous increase in oxidative metabolism. Since the cell density of cells treated with FGF-2, as observed in phase-contrast pictures, was similar to that of other tested conditions, the decrease in cell impedance could be linked to a reduction in cell adherence rather than a decrease in cell number. This could represent a strategic trade-off of the cells, aiming to allocate resources towards energy production under the GF stimulation at the expense of maintaining strong adhesion to the substrate. The influence of FGF-2 was concentration-dependent, with 50 ng/mL inducing a stronger effect. Furthermore, the addition of FGF-2 increased the energy potential of the cells compared to 2% FBS. Both mitochondrial respiration and glycolytic metabolism were balanced, mirroring the metabolism of cells cultured in 10% FBS and identifying FGF-2 as a promising candidate for use as an additive for cultivating AOXlar7y cells, although further optimization is required.

Insulin-like growth factor-1 (IGF-1) is a well-characterized GF with both mitogenic and pleiotropic properties, promoting cell proliferation, survival, and also development, differentiation through activation of key signaling pathways such as PI3K/AKT and MAPK/ERK as well as metabolism (Khan et al., 2025). In the osteoblast primary culture of Gilthead bream (*Sparus aurata*), IGF-1 has been linked with promoting proliferation (Capilla et al., 2011). Similarly to FGF-2, it induced a marked decrease in cell impedance of AOXlar7y cells while inducing a drastic increase in cell metabolism. These results support the hypothesis of cell adherence impairment rather than cell number reduction, especially considering the cell density observed in phase-contrast images, which was similar to that of 10% FBS. Already, the low concentration of IGF-1 was able to mimic the basal oxygen consumption observed in cells incubated with 5% FBS. In comparison, 50 ng/mL reached 10% FBS oxygen consumption levels. However, while the maximum respiration capacity was slightly increased compared to cells cultivated with 2% FBS, the spare respiratory capacity appeared to be compromised, especially when compared to cells cultivated with 10% FBS. Under metabolic stress conditions, IGF-1-treated cells preferentially utilize glucose anaerobically. This is unsurprising since IGF-1 is involved in glucose uptake and is associated with glycolysis and lipogenesis (Montserrat et al., 2012; Kasprzak, 2021). However, a previous study analyzing glycolysis and glycolytic capacity of the AOXlar7y cells suggested a minor role of glycolysis in these cells when cultured with 10% FBS (Lutze et al., 2024). This indicates that the metabolic responsiveness of AOXlar7y cells to IGF-1 may be context-dependent. Further studies are needed to gain a deeper understanding of the role of IGF-1 in AOXlar7y cells.

Overall, IGF-1 (50 ng/mL) was the best candidate among the GFs, CKs and concentration tested as it was able to mimic cell proliferation and metabolism at a level comparable to 10% FBS. However, the impaired reserve respiratory capacity and the shift towards glycolytic metabolism indicate that IGF-1 cannot be used alone in the serum-reduced medium. Instead, multiple factors should be used to modulate adhesion, proliferation, and metabolism accordingly to achieve the properties of cells cultivated with 10% FBS. In addition, the impaired cell adherence observed in the cell impedance analysis represents another important limitation that may constrain downstream applications, highlighting the need for further medium optimization in this regard. Another possible candidate might be LIF, which was found to promote oxidative metabolism in AOXlar7y cells. A comparative analysis of the 10% FBS medium before and after cell cultivation, as demonstrated by O'Neill et al. (2022) could provide valuable insights into AOXlar7y cultivation needs, especially considering that cells actively secrete factors into the medium (Mukherjee and Mani, 2013). Moreover, as shown in previous studies, an adaptation phase (Radošević et al., 2016) and repeated administration of GFs (Lotz et al., 2013) can potentially alter the cellular responses. However, since little is currently known about the metabolism of sturgeon larval cells, it remains challenging to assess how well the *in vitro* responses reflect actual *in vivo* conditions. Although 10% FBS was used as a reference in the present study, the high serum concentration—and the resulting elevated metabolic and proliferation rates—may not be fully representative of the physiological state of sturgeon larval cells *in vivo*. Building on previous work, Jozef et al., 2025 developed a systematic and transparent approach to create a fully defined, serum-free medium formulation. As a reference model, they used the well-established rainbow trout gill cell line RTgill-W1 cell line (Bols et al., 1994), which was already adapted to grow with 5% FBS instead of the traditional 10%. Starting from L-15 medium as the basal component, they selectively added key compound groups typically present in animal serum - such as hormones, proteins, vitamins, metals, minerals, lipids–to identify the minimal yet sufficient combination for supporting vertebrate cell growth. Due to the epithelial origin of the RTgill-W1 cells, epidermal growth factor (EGF) and hydrocortisone were included early on. Based on available literature and known serum composition, they tested 27 components (13 of which came from a lipid mixture), applying both direct and gradual adaptation strategies. Their formulation successfully supported long-term growth and showed chemical sensitivity profiles comparable to cells cultured with FBS. Importantly, they emphasized that verifying toxicological responses in the adapted cells is critical to preserve the correlation between *in vivo* to *in vitro* models. In contrast, our current formulation did not include several essential factors used by Jozef et al., such as albumin, vitamin B12, insulin, transferrin, selenium or lipids, which may explain the comparatively lower performance of the tested GFs and CKs under serum-reduced conditions. Despite this, our study explored a broader range of signaling molecules (e.g., GFs and CKs), which were only limitedly addressed by Jozef et al. (2025). By integrating Jozef et al. defined medium components - especially the critical hormones and lipid mixtures-with the functional GFs and CKs identified in our present study, it is now feasible to propose a first chemically-defined animal-free formulation tailored for AOXlar7y cells. Future steps should include systematic testing of this combined approach and evaluation of chemical sensitivity to validate the *in vitro* relevance.

## 5 Conclusion

In conclusion, a fully functional, serum-free medium for cultivating AOXlar7y cells has not yet been achieved. However, this study highlights the critical role of serum in maintaining energy production and supporting proliferation in these sturgeon cells. None of the individual GF or CK could fully compensate for the effects of serum reduction. Nevertheless, our results demonstrated the cell line’s potential to adapt to serum-reduced conditions in the short term and the presence of fine-tuning metabolic adaptations in response to the different GFs and CKs. Given the evolutionary conservation of molecular pathways among vertebrates, commercially available mammalian recombinant GFs and CKs were able to affect the sturgeon cell behavior. However, some GFs and CKs are more conserved across species than others, and even minor differences in their structure can affect the cellular responses. Currently, species-specific medium components are either unavailable or unaffordable commercially. Their evaluation is essential to better reflect the species’ physiology *in vitro* and potentially obtain a more effective result. Future work should explore combinatorial applications of multiple GFs and CKs at optimized concentrations, complemented by essential additives such as lipids, vitamins, and trace elements. Incorporating additional functional assays and extending the duration of experiments will be key to understanding the role of these factors in proliferation, differentiation, and long-term adaptation. Overall, this study lays the foundation for the stepwise replacement of animal-derived components in sturgeon cell culture and represents an important advance toward sustainable, species-appropriate *in vitro* systems for aquaculture and environmental research.

## Data Availability

The raw data supporting the conclusions of this article will be made available by the authors, without undue reservation.
